# Nodular Histiocytic Aggregates of the Greater Omentum Mimicking Peritoneal Carcinomatosis on Imaging

**DOI:** 10.7759/cureus.70063

**Published:** 2024-09-24

**Authors:** Nada Elmukhtar, Jawad Al-Khalaf, Ali Alsehaiw, Lobaina Abozaid

**Affiliations:** 1 Department of Pathology, King Fahad Hospital, Hofuf, SAU; 2 Department of Radiology, King Fahad Hospital, Hofuf, SAU; 3 Department of Pathology, Qassim University, Buraidah, SAU

**Keywords:** greater omentum, histiocytic aggregates, hiv, mesothelial hyperplasia, peritoneal carcinomatosis

## Abstract

Nodular histiocytic/mesothelial hyperplasia (NHMH) is a benign reactive process characterized by the proliferation of histiocytic cells and scattered mesothelial cells. We report a case of NHMH of the greater omentum in a 56-year-old HIV-positive male who presented with paraumbilical swelling and multiple omental nodules on imaging. The preoperative assessment of the patient reveals an inverted CD4/CD8 ratio. The patient underwent herniorrhaphy and an excisional biopsy of the greater omentum. Pathological examination reveals mesothelial-lined fibroadipose tissue containing nodular aggregates of histiocytic cells, with immunohistochemical staining for CD68 and CK7 confirming the histiocytic and mesothelial nature of the lesional cells, respectively. The patient received antiretroviral therapy and antibiotics and was discharged in good condition.

This is the first case report presenting the occurrence of NHMH in an HIV-positive patient. Although it is a benign lesion, it can mimic malignancy on imaging. The diagnosis was made based on histopathological examination along with immunohistochemical staining. In our case report, NHMH presents with diffuse infiltration of the greater omentum on imaging. This emphasizes the importance of recognizing this pathological entity along with clinicopathological correlation to avoid misdiagnosis that may adversely affect patient outcomes.

## Introduction

Nodular histiocytic/mesothelial hyperplasia (NHMH) is a benign reactive process characterized by nodular histiocytic proliferation and scattered mesothelial cells [1]. The lesion was described by Rosai and Dehner in 1975 in a hernia sac, without the use of electron microscopy or immunohistochemistry [2]. NHMH has commonly been identified on surfaces lined by mesothelium, including the omentum, pleura, and pericardium, as well as in non-mesothelial lined organs such as the lung, inguinal region, urinary bladder, and pelvic cavity. Microscopically, NHMH consists of sheets of mesothelial cells and histiocytes within a fibrin meshwork, with or without an intervening vascular network. These lesions are noteworthy because they may be mistaken for malignancies both radiologically and pathologically [3-4]. We report a case of NHMH of the greater omentum in a 56-year-old HIV-positive male who presented with umbilical swelling and multiple omental nodules, which mimicked peritoneal carcinomatosis on imaging.

## Case presentation

A 56-year-old HIV-positive male patient presented with umbilical swelling; on examination, blood pressure was 120/70 mmHg, heart rate was 82 beats per minute, respiratory rate was 15 breaths per minute, and temperature was 37°C. There was no pallor, jaundice, or lymphadenopathy. On chest examination, abdominal CNS examination was unremarkable. The patient underwent surgical herniorraphy of umbilical hernia and excisional biopsy from the greater omentum.

**Figure 1:**
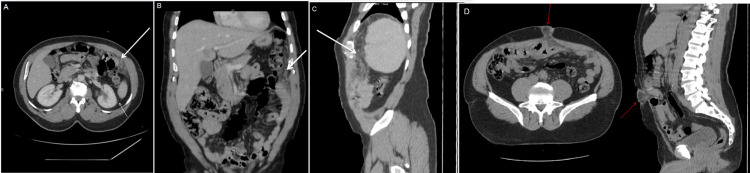
CT scan of the abdomen after IV contrast administration (A) axial, (B) coronal, and (C) sagittal reformatted images demonstrate the greater omentum is infiltrated by linear and nodules of soft tissue at the left upper abdomen (misty omentum), white arrows. Note: Clear appearance of normal intra-abdominal fat (thin white arrow). (D) There is fat-containing umbilical hernia with fat stranding. The hernial neck measures about 1.5 cm, and the hernial sac measures about 3.6 x 2.3 cm CT: computed tomography, IV: intravenous

Upon surgical admission, laboratory test results for the CBC are found in Table 1, and the microbiology workup is presented in Table 2. Flow cytometry analysis shows a markedly inverted CD4/CD8 ratio due to a moderately increased CD8 absolute count with a relatively decreased CD4 count. NK and B cells are preserved. Increased expression of HLA-DR on CD8 T cells denotes the progression of HIV. CT of the abdomen reveals diffusely infiltrated greater omentum by soft tissue nodules with fat stranding and increased attenuation (misty appearance), as shown in Figure 1 (A, B, C). This finding is associated with several processes, including infiltration by tumors, inflammatory cells, edema, and fibrosis. The radiological differential diagnosis includes peritoneal carcinomatosis, peritoneal lymphomatosis, and tuberculous peritonitis. Additionally, there is a fat-containing umbilical hernia with fat stranding, as seen in Figure 1 (D).

**Table 1:** CBC laboratory result upon admission WBC: white blood cells, RBC: red blood cells, HGB: hemoglobin, HCT: hematocrit, MCV: mean corpuscular volume, MCH: mean corpuscular hemoglobin, MCHC: mean corpuscular hemoglobin concentration, RDW-SD: red cell distribution width standard deviation, RDW-CV: red cell distribution width coefficient of variation

Component	Result	Reference range
WBC (X10^9^/L)	5.95	4-11
RBC (x10^12^ L)	5.46	5-7
HGB (g/dL)	14.4	13-17
HCT (%)	43.5	37-51
MCV (fL)	79.7	80-94
MCH (Pg)	26.4	27-31
MCHC (g/dL)	33.1	33-37
Platelet (X 10^9^L)	225	150-400
RDW-SD (fL)	41.3	39-46
RDW-CV (%)	14.4	11.5-15.5

**Table 2:** Microbiology laboratory result TB: tuberculosis, DNA: deoxyribonucleic acid, AFB: acid-fast bacilli, MGIT: mycobacteria growth indicator tube, L-J: Löwenstein–Jensen, IgG: immunoglobulin G, IgM: immunoglobulin M, anti-HCV: anti-hepatitis C virus, HIV: human immunodeficiency virus

Test	Result
Direct molecular TB test (DNA): omental biopsy	Negative
AFB stain: omental biopsy	No AFB seen
MGIT culture	No AFB isolated after 6 weeks
L-J culture	No AFB isolated after 6 weeks
Blood culture	Positive cocci
Urine culture	No growth
Sputum culture	Normal flora
Toxoplasma antibodies IgG	Negative
Toxoplasma antibodies IgM	Negative
Hepatitis B surface antigen	Negative
Anti-HCV	Negative
HIV confirmatory test (western blot)	Positive

**Figure 2:**
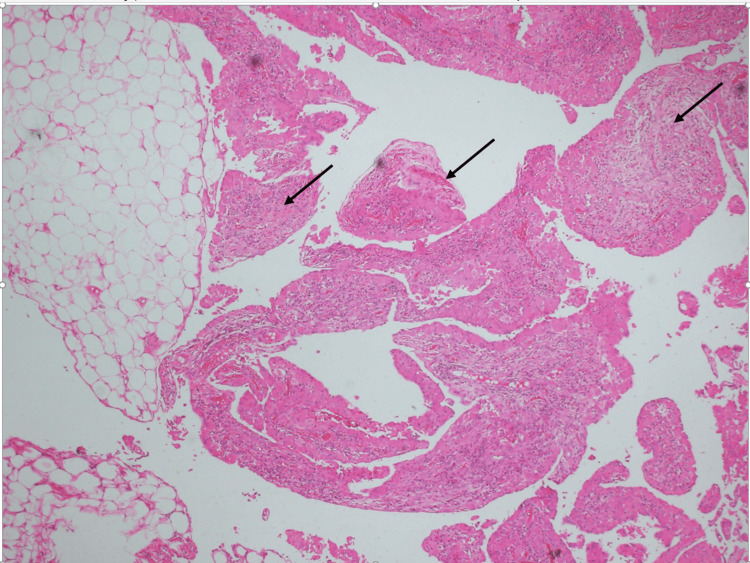
Nodular aggregates of histiocytic cells (H&E stain 20x) H&E: hematoxylin and eosin

**Figure 3:**
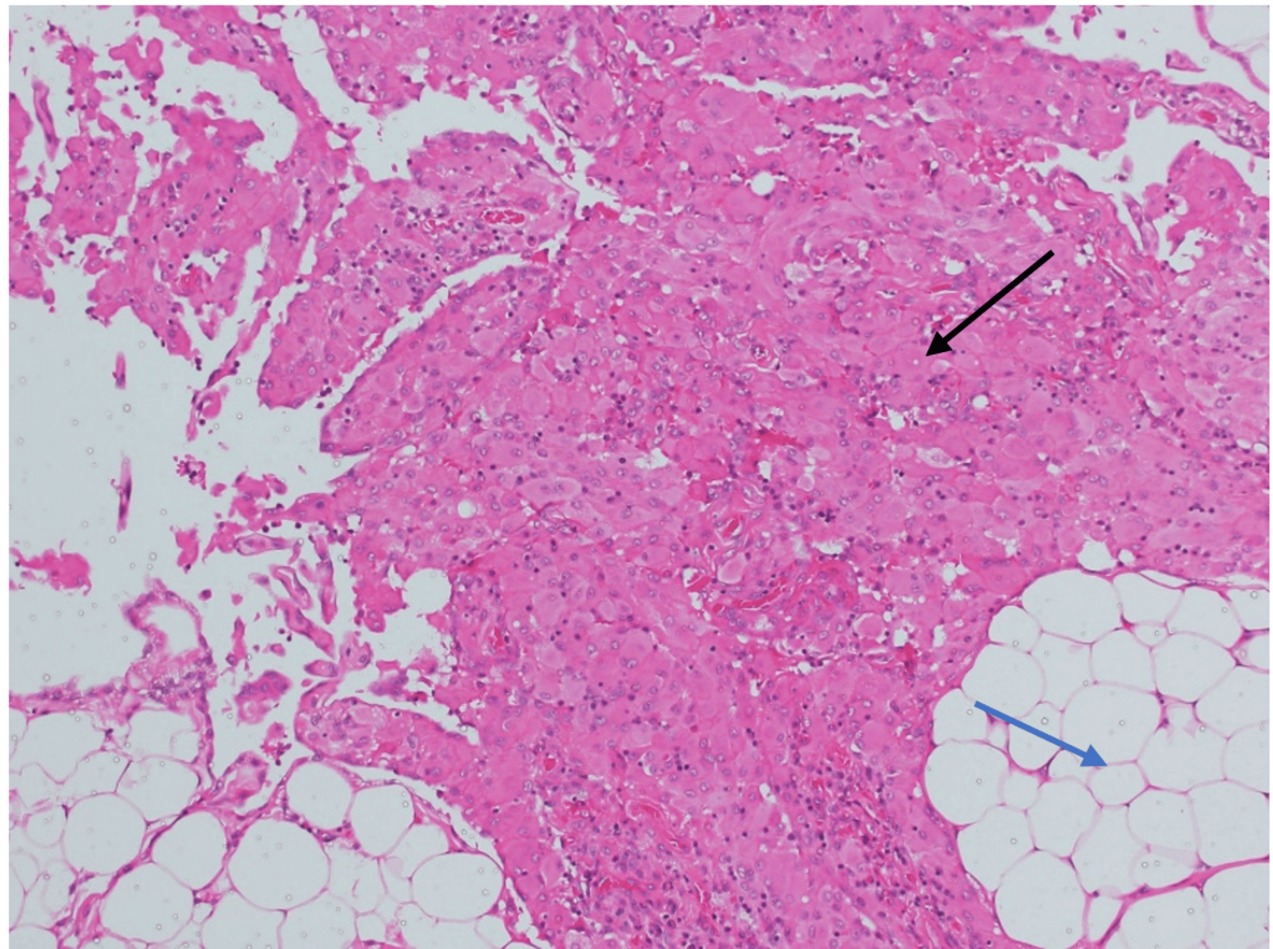
H&E stain 10x: Microscopic examination reveals nodular aggregates of histiocytic cells with eosinophilic cytoplasm (black arrow) embedded in fibroadipose stroma (blue arrow) H&E: hematoxylin and eosin

The macroscopic examination of the surgically removed specimen described an irregular rectangular piece of fatty tissue with congested blood vessels, measuring 13.5 × 7.5 × 3 cm. Histological examination (Figures 2-3) reveals vascularized fibroadipose tissue showing sheets and aggregates of large histiocytic cells with round-ovoid nuclei, some with distinct nucleoli, and abundant eosinophilic cytoplasm intermixed with inflammatory cells dominated by plasma cells, along with occasionally interspersed eosinophils and a few multinucleated giant cells. No granulomas, necrosis, or malignancy were observed.

**Figure 4:**
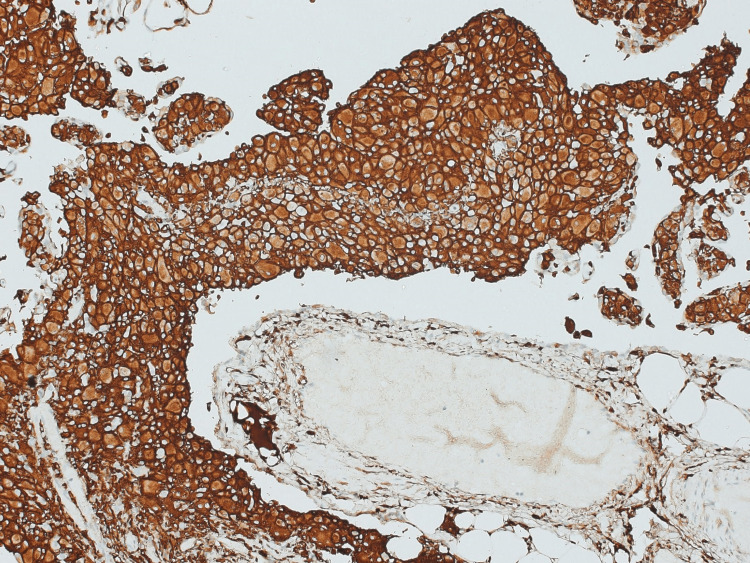
Histiocytic cells showing strong diffuse staining for CD163 (10x)

**Figure 5:**
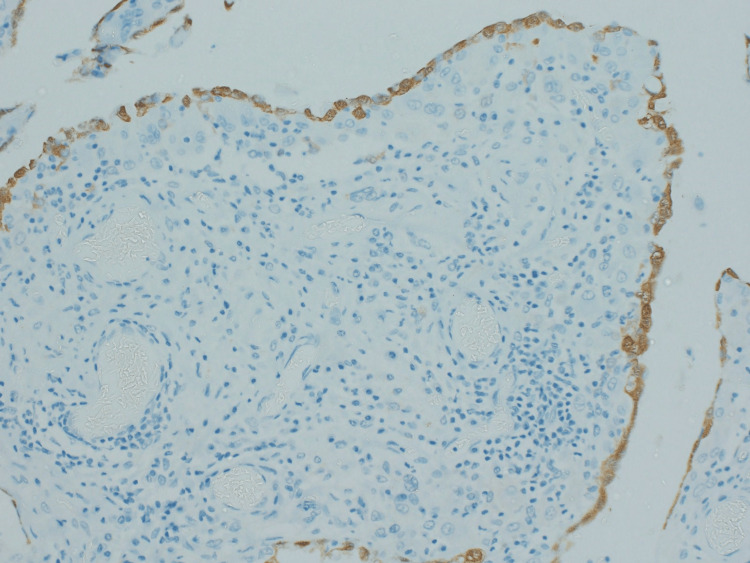
Calretinin staining highlighting the mesothelial lining of the omentum (20x)

**Figure 6:**
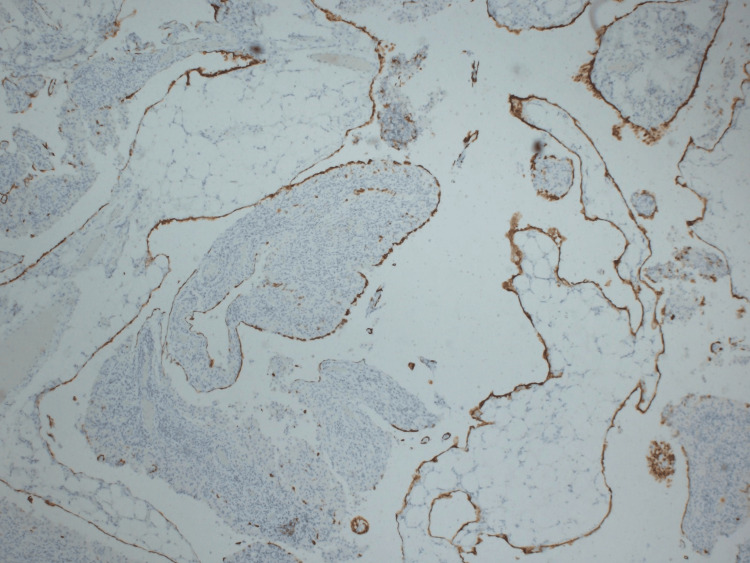
Mesothelial cells showing cytoplasmic staining for CK7 (4x)

Immunohistochemical staining on paraffin-embedded sections with adequate internal control was performed and revealed histiocytic cells that were immunoreactive for CD163, exhibiting a strong and diffuse pattern of staining (Figure 4). Calretinin staining highlighted the mesothelial lining of the omentum (Figure 5), and CK7-immunoreactive mesothelial cells were also observed (Figure 6).

## Discussion

This case involves a 56-year-old male HIV-positive patient with NHMH, which represents a rare occurrence in HIV-positive individuals. NHMH is a reactive process characterized by the non-neoplastic proliferation of histiocytes and mesothelial cells [5-6]. There are no characteristic clinical manifestations, and it is usually an incidental finding. The age of presentation ranges from four to 85 years, with a mean age of presentation between 40 and 50 years. However, the disease has also been reported in children and young adults. Reported cases in the literature show that there is no sex predilection for the lesion. A study by Chung et al. indicated that the lesion is slightly more prevalent in females [3]; however, a study by Michal et al. reported that the disease is more common in females, with a female-to-male ratio of 2:1 [4].

NHMH is most commonly found on mesothelial-lined surfaces, namely the pleura, peritoneum, and pericardium. The most common anatomical site for NHMH is the peritoneum, followed by the skin, thymus, pleura, pericardium, and mediastinum (Table 3) [4,5,7]

**Table 3:** Distribution of the lesion according to age and sex

Number	Author	Year reported	Site	Age	Sex
1	Rosai and Dehner [5]	1975	Peritoneum	41 years	Male
2			Peritoneum	8 years	Male
3			Peritoneum	8 years	Male
4			Peritoneum	6 weeks	Male
5			Peritoneum	57 years	Male
6			Peritoneum	6 weeks	Male
7			Peritoneum	10 years	Male
8			Peritoneum	22 years	Female
9			Peritoneum	6 years	Male
10			Peritoneum	2 years	Male
11			Peritoneum	1 year	Male
12			Peritoneum	84 years	Female
13			Peritoneum	18 months	Male
14 Present case			Peritoneum	56 years	Male
15	Chung [3]	2016	Peritoneum	38 years	Female
16	Michal [4]	2016	Thyroid	55 years	Female
17			Thyroid	37 years	Female
18	Suarez-Vilela [8]	2002	Peritoneum	71 years	Male
19	Chikkamuniyappa [9]	2004	Peritoneum	36 years	Female
20			Peritoneum	37 years	Female
21	Yang [1]	2012	Peritoneum	44 years	Female
22			Peritoneum	6 years	Female
23			Peritoneum	41 years	Female
24	Chen [10]	2017	Spermatic cord cyst	4 years	Male
25	Nicolas [11]	2011	Pericardial	60 years	Male
26	Chikkamuniyappa [9]	2004	Pericardial	47 years	Female
27	Mallick [12]	2016	Cardiac valve	32 years	Female
28	Chikkamuniyappa [9]	2004	Pleural	53 years	Female
29			Pleural	Elder	Male
30	Chan [7]	1997	Lung	57 years	Male
31			Lung	51 years	Female
32	Rossi [13]	2007	Lung	79 years	Male
33	Michal [4]	2016	Skin	68 years	Male
34			Skin	85 years	Male
35			Skin	63 years	Male
36			Skin	48 years	Male
37			Skin	79 years	Male
38			Skin	84 years	Female
39			Skin	85 years	Female
40			Skin	78 years	Female
41			Skin	29 years	Female

The pathophysiology underlying NHMH is unknown. However, it occurs as a consequence of an unspecific reaction to an injury (e.g., trauma, inflammation, infiltrating malignancy, and surgical procedure) [4]. It has been suggested that the nodular aggregation of these histiocytes and mesothelial cells is mediated by adhesion molecules [5]. However, this lesion was found in organs that are devoid of a mesothelium, such as the endocardium, and inside a dissecting aneurysm. A possible explanation for that is the accidental insertion of mesothelial cells by a variety of procedures. These mesothelial cells, once inside the vascular space, lead to mechanical irritation, resulting in histiocytic proliferation. Mesothelial cells express CD34 and other adhesion molecules such as ICAM-1 and VCAM-1, which mediate the adhesion of mesothelial cells to histiocyte/monocyte elements by the L-selectin [12,14].

NHMH is reported to be found in association with a variety of diseases, including ovarian cancer, chronic lymphocytic leukemia, and antiphospholipid syndrome. This lesion is very rare, and, to the best of our knowledge, this is the first reported case that is associated with HIV infection [4].

Michal et al. published a study of 50 cases that described Langerhans cell histiocytosis (LCH) as a differential diagnosis of NHMH. LCH comprises histiocytes with unique oval-shaped grooved nuclei associated with eosinophilic infiltration, along with the expression of CD1a by histiocytes. Additionally, the proliferating histiocytes in NHMH may exhibit cytological atypia that could be misinterpreted as malignancies [4]. Furthermore, Chan et al. reported two cases of NHMH in transbronchial biopsies that were initially considered to be neuroendocrine neoplasms or meningiomas [7]. Chung et al. reported a case of pelvic nodular histiocytic aggregates/mesothelial hyperplasia in a patient with endometriosis and leiomyoma [3].

## Conclusions

NHMH is a benign lesion that should be considered in the differential diagnosis of all diffuse nodular masses in mesothelial-lined tissue. The diagnosis of NHMH is primarily based on histopathological examination, supported by immunohistochemical staining. In this study, we document the occurrence of NHMH in an HIV-positive patient. In our case report, NHMH presented with soft tissue infiltration of the greater omentum, exhibiting an appearance that mimicked peritoneal carcinomatosis on imaging. Awareness of this pathological entity is crucial to avoid erroneous diagnoses and aggressive, unnecessary management, which can negatively affect overall patient outcomes and prognosis. Accurate diagnosis is achieved through adequate histopathological assessment and clinicopathological correlation.
